# Prevalence of metabolic syndrome risk factors and their relationships with renal function in Chinese centenarians

**DOI:** 10.1038/s41598-018-28316-x

**Published:** 2018-06-29

**Authors:** Shihui Fu, Yao Yao, Fuxin Luan, Yali Zhao

**Affiliations:** 1 Department of Geriatric Cardiology, Chinese People’s Liberation Army General Hospital, Beijing, China; 2 Department of Cardiology and Hainan Branch, Chinese People’s Liberation Army General Hospital, Beijing, China; 3 Institute of Geriatrics, Chinese People’s Liberation Army General Hospital, Beijing, China; 4 Beijing Key Laboratory of Aging and Geriatrics, Chinese People’s Liberation Army General Hospital, Beijing, China; 5 Central Laboratory, Hainan Branch of Chinese People’s Liberation Army General Hospital, Sanya, China

## Abstract

As the first time, this study was to investigate the prevalence of metabolic syndrome (MetS) risk factors and explore their relationships with renal function in Chinese centenarians. China Hainan Centenarian Cohort Study was performed in 18 cities and counties of Hainan Province. Home interview, physical examination and blood analysis were performed in 874 centenarians following standard procedures. Prevalence of MetS was 15.6% (136 centenarians). There were 229 centenarians with abdominal obesity (26.2%), 645 centenarians (73.8%) with hypertension, 349 centenarians with dyslipidemia (39.9%) and 92 centenarians with diabetes mellitus (10.5%). In multivariate linear regression, age, smoking, waist circumstance (WC), systolic blood pressure (SBP) and triglyceride levels were inversely and diastolic blood pressure (DBP) levels were positively associated with glomerular filtration rate levels (P < 0.05 for all). This study reported low prevalence of MetS risk factors and demonstrated that age, smoking, abdominal obesity (WC), hypertension (SBP and DBP) and triglyceride levels were independently associated with renal function in Chinese centenarians. This study provided reliable data about Chinese centenarians, analyzed significant relationships between Mets risk factors and renal function, and explained possible reason (low prevalence of MetS and its risk factors) and mechanism (interrelationship of age, Mets risk factors with renal function) of longevity.

## Introduction

During the last few decades, metabolic syndrome (MetS) and renal function decline (RFD) continue to grow in prevalence all over the world, and this trend is particularly obvious in developing countries^1^. MetS refers to a clustering of risk factors: abdominal obesity, hypertension, dyslipidemia and diabetes mellitus (DM), all which contribute to the development of cardiovascular disorders and events^2–4^. Meanwhile, RFD plays a significant role in the progression of cardiovascular disorders, and has close association with cardiovascular events^5,6^. More importantly, both MetS and RFD have obvious effects on mortality, and accelerate the occurrence of adverse outcome^7,8^. Previous studies have observed the prevalence of MetS and analyzed their relationships with renal function, but drawn controversial conclusions^8,9^. Meanwhile, most of these studies were performed in the general population of developed countries, and there may be obviously different conclusions in the centenarians in China^9^. Moreover, different conclusions may also derive from race, and thus it is very essential to perform this study in Chinese^10^.

The centenarians have been suggested to have a delayed or escaped onset and interaction of age-related illnesses, such as MetS and RFD^11^. Some centenarians may experience a delayed onset of age-related illnesses (delayers), while others may do not succumb to any age-related illnesses (escapers)^12^. Thus, the centenarians may represent a prototype of successful aging^13^. However, it is still under scientific debate^14^. More importantly, what is this model of successful aging? Studies analyzing this model in the centenarians could provide valuable information for early promoting successful aging and preventing age-related diseases. As a possible part of this model, whether the interaction between MetS and RFD exists in the aging process of centenarians is still unclear and needs further studies.

Prevalence of MetS increases with age, reaching 42.0% in U.S. adults 70 years or older^15^. However, prevalence of MetS and its risk factors are still unclear in Chinese centenarians^16^. Moreover, its relationship with renal function still has significant debate in the elderly, not to say the centenarians^17^. Considering the specificity of centenarians, previous studies in the general population can not accurately represent the centenarians^18^. Meanwhile, in order to understand the reasons and mechanisms of longevity, it is very valuable to perform the studies in Chinese centenarians. Hainan is a longevity area with the highest population density of centenarians in China, and China Hainan Centenarian Cohort Study (CHCCS) with a considerable sample size provides a significantly population-based sample of Chinese centenarians. As the first time all over the world, this study was designed to investigate the prevalence of MetS risk factors and explore their relationships with renal function in a representative sample of Chinese centenarians.

## Results

For all centenarians, median age was 102 (100–115) years, and males account for 18.9%. Prevalence of MetS was 15.6% (136 centenarians). For MetS risk factors, there were 229 centenarians with abdominal obesity (26.2%), 645 centenarians (73.8%) with hypertension, 349 centenarians with dyslipidemia (39.9%) and 92 centenarians with DM (10.5%). There were 371 centenarians with GFR <60 ml/min/1.73 m^2^ (42.4%). As shown in Table 1, the centenarians with GFR <60 ml/min/1.73 m^2^ tended to be smoking and have MetS, abdominal obesity and hypertension than those with GFR ≥60 ml/min/1.73 m^2^ (P < 0.05 for all). There were significantly more participants with higher WC, SBP, TG levels and lower HDL-C levels in the centenarians with GFR <60 ml/min/1.73 m^2^ than those with GFR ≥60 ml/min/1.73 m^2^ (P < 0.05 for all). MetS had significant relationship with GFR (r = −0.127, P < 0.001; EXP(β): 1.971, 95% CI: 1.362–2.853, P < 0.001; standard β: −0.112, P = 0.001). The number of MetS risk factors had significant relationship with GFR (r = −0.106, P = 0.002; EXP(β): 1.222, 95% CI: 1.074–1.390, P = 0.002; standard β: −0.094, P = 0.005). In the simple correlation analyses (Table 2), smoking, abdominal obesity, WC, TG and HDL-C levels were significantly related to GFR levels (P < 0.05 for all). SBP (P = 0.070) and DBP (P = 0.054) were moderately but not significantly related to GFR levels. In the multivariate linear regression analysis (Table 3), age, smoking, WC, SBP and TG levels were inversely and DBP levels were positively associated with GFR levels (P < 0.05 for all). In the multivariate logistic regression analysis (Table 4), smoking, abdominal obesity and hypertension were independently associated with GFR <60 ml/min/1.73 m^2^ (P < 0.05 for all).

**Table 1:** Prevalence of metabolic syndrome risk factors and description of other characteristics in centenarians.

Characteristics	Total (n = 874)	GFR <60 ml/min/1.73 m^2^ (n = 371)	GFR ≥60 ml/min/1.73 m^2^ (n = 503)	P value
Age (year)	102 (101–104)	102 (101–104)	102 (101–104)	0.495
Males (%)	165 (18.9)	75 (20.2)	90 (17.9)	0.386
Smoking (%)	29 (3.3)	21 (5.7)	8 (1.6)	0.001
MetS (%)	136 (15.6)	77 (20.8)	59 (11.7)	<0.001
Abdominal obesity (%)	229 (26.2)	115 (31.0)	114 (22.7)	0.006
Hypertension (%)	645 (73.8)	288 (77.6)	357 (71.0)	0.027
Dyslipidemia (%)	349 (39.9)	151 (40.7)	198 (39.4)	0.690
DM (%)	92 (10.5)	37 (10.0)	55 (10.9)	0.647
WC (cm)	75 (70–80)	76 (71–82)	74 (68–80)	0.007
SBP (mmHg)	150 (136–170)	152 (138–172)	148 (133–167)	0.008
DBP (mmHg)	75 (67–83)	74 (67–84)	76 (67–83)	0.549
TC (mmol/L)	4.58 (3.99–5.27)	4.54 (3.96–5.18)	4.62 (4.03–5.31)	0.142
TG (mmol/L)	1.03 (0.80–1.41)	1.11 (0.84–1.47)	0.98 (0.77–1.33)	<0.001
HDL-C (mmol/L)	1.40 (1.17–1.67)	1.35 (1.13–1.62)	1.43 (1.20–1.70)	0.014
LDL-C (mmol/L)	2.72 (2.27–3.26)	2.65 (2.26–3.24)	2.75 (2.29–3.27)	0.236
FBG (mmol/L)	4.82 (4.20–5.75)	4.97 (4.22–5.76)	4.75 (4.18–5.72)	0.168
GFR (ml/min/1.73 m^2^)	63.11 (52.34–73.39)	50.48 (43.82–55.13)	71.70 (65.97–79.94)	<0.001

Abbreviations: GFR: glomerular filtration rate; MetS: metabolic syndrome; DM: diabetes mellitus; WC: waist circumstance; SBP: systolic blood pressure; DBP: diastolic blood pressure; TC: total cholesterol; TG: triglyceride; HDL-C: high-density lipoproteincholesterol; LDL-C: low-density lipoprotein cholesterol; FBG: fasting blood glucose.

**Table 2:** Relationships between metabolic syndrome risk factors and GFR in simple correlation analyses.

Characteristics	r	P value
Age (year)	−0.043	0.199
Females/males	0.026	0.449
Smoking	−0.079	0.020
Abdominal obesity	−0.096	0.005
Hypertension	−0.045	0.183
Dyslipidemia	−0.029	0.389
DM	0.022	0.524
WC (cm)	−0.157	<0.001
SBP (mmHg)	−0.061	0.070
DBP (mmHg)	0.065	0.054
TC (mmol/L)	0.039	0.247
TG (mmol/L)	−0.121	<0.001
HDL-C (mmol/L)	0.067	0.047
LDL-C (mmol/L)	0.036	0.291
FBG (mmol/L)	−0.036	0.294

Abbreviations: GFR: glomerular filtration rate; DM: diabetes mellitus; WC: waist circumstance; SBP: systolic blood pressure; DBP: diastolic blood pressure; TC: total cholesterol; TG: triglyceride; HDL-C: high-density lipoproteincholesterol; LDL-C: low-density lipoprotein cholesterol; FBG: fasting blood glucose.

**Table 3:** Relationships between metabolic syndrome risk factors and GFR in multivariate linear regression analysis.

Characteristics	Standard β	t	Standard error	P value
Age (year)	−0.071	−2.129	0.002	0.034
Females/males	0.008	0.215	0.011	0.829
Smoking	−0.070	−2.029	0.024	0.043
WC (cm)	−0.193	−5.578	<0.001	<0.001
SBP (mmHg)	−0.086	−2.102	<0.001	0.036
DBP (mmHg)	0.132	3.205	<0.001	0.001
TC (mmol/L)	0.105	0.803	0.016	0.422
TG (mmol/L)	−0.083	−1.985	0.008	0.047
HDL-C (mmol/L)	−0.048	−0.855	0.018	0.393
LDL-C (mmol/L)	−0.017	−0.150	0.018	0.880
FBG (mmol/L)	0.014	0.411	0.003	0.681

Abbreviations: GFR: glomerular filtration rate; WC: waist circumstance; SBP: systolic blood pressure; DBP: diastolic blood pressure; TC: total cholesterol; TG: triglyceride; HDL-C: high-density lipoproteincholesterol; LDL-C: low-density lipoprotein cholesterol; FBG: fasting blood glucose.

**Table 4:** Relationships between metabolic syndrome risk factors and GFR in multivariate logistic regression analysis.

Characteristics	EXP(β)	95% confidence interval	P value
Age (year)	1.021	0.972–1.073	0.406
Females/males	0.941	0.653–1.358	0.746
Smoking	3.980	1.681–9.424	0.002
Abdominal obesity	1.566	1.143–2.145	0.005
Hypertension	1.396	1.016–1.918	0.040
Dyslipidemia	0.990	0.747–1.314	0.947
DM	0.894	0.571–1.400	0.625

Abbreviations: GFR: glomerular filtration rate; DM: diabetes mellitus.

## Discussion

Studies about the centenarians can help us understand the reasons and mechanisms of longevity. In previous studies, Mets has a prevalence more than 20% in the general population and a higher prevalence in the elderly^19–21^. In U.S. adults, prevalence of MetS increases with age, reaching 42.0% in those 70 years or older^15^. This study reported that there was obviously low prevalence of Mets (15.6%) and its risk factors in Chinese centenarians. Georgia Centenarian Study has concluded that major barriers to reaching centenarians come from several incident chronic age-related disorders, especially cardiovascular disorders^20^. Meanwhile, Mets and its risk factors play significant roles in the development of cardiovascular and other chronic age-related disorders. Moreover, longevity data from Framingham Study have supported that MetS risk factors, such as blood pressure, glucose and lipids, are significant contributors to morbidity and mortality^22^. Thus, low prevalence of Mets and its risk factors found in this study was a possible reason of longevity in Chinese centenarians.

Both MetS and RFD have been underlined to be independently associated with cardiovascular incidence, events and mortality^2–8^. Previous studies have shown significant relationship between MetS and renal function, but drawn controversial conclusions^8,9,17^. Meanwhile, most of these studies about the centenarians are performed in the general population of developed countries, with little information available on the centenarians in China^9,18^. Additionally, the data on ethnic Chinese are limited, and the relationship between MetS and renal function is still unknown in ethnic Chinese^9,10^. This study realized a possible mechanism of longevity in Chinese centenarians that not only age, but also Mets and its risk factors had significant relationships with renal function. On the one hand, Mets and its risk factors may impair the renal function, aggravate the cardiovascular disorders and reduce the life expectancy. The hypothesized mechanism is that as age increases, the clustering of MetS risk factors may result in oxidative stress and endothelial dysfunction, which consequently cause the atherosclerosis-related RFD^23^. In turn, RFD may gradually affect the blood pressure, glucose and lipids, and also aggravate the metabolic disturbance and promote the development of MetS^24^. On the other hand, the relationships between age, Mets and renal function also remind us that Mets and RFD may result from a common cause linked to the aging phenomenon. Either one of MetS and RFD may enforce the effects of other one on cardiovascular disorders and their incidence, and the interrelationship between MetS and RFD may worsen further cardiovascular events and mortality^7,8^.

Abdominal obesity is a fundamental pathology of MetS, and contributes to the development of other MetS risk factors, such as hypertension, dyslipidemia and DM. Abdominal obesity is significantly associated with high mortality risk, and there is an obviously increased prevalence of abdominal obesity in many developed and developing countries^25,26^. There have been inconsistent study results on the relationship between abdominal obesity and renal function. Several studies have realized that abdominal obesity is significantly associated with renal function in the general population. But other study has not verified significant association between abdominal obesity and renal function in the elderly^16^. This study observed that with a prevalence of 26.2%, abdominal obesity was independently associated with renal function in Chinese centenarians.

As prevalence of hypertension is rapidly increasing all over the world, the relationship between blood pressure and renal function has drawn considerable attention. Previous studies have shown that hypertension was significantly associated with renal function^27,28^. However, other study has also found that blood pressure has no significant association with renal function^16^. This study showed that with a prevalence of 73.8%, hypertension was inversely associated with renal function. More interestingly, SBP was inversely associated with renal function, but DBP was positively associated with renal function in this study. One the one hand, elevated SBP may induce the glomerulosclerosis and lower GFR, while reduced DBP may cause the renal hypoperfusion and lower GFR. On the other hand, RFD may affect the sodium and water retention, activate the renin-angiotensin-aldosterone system and induce the abnormality of SBP and DBP^29^.

It is a significant issue to analyze the relationships between different types of dyslipidemia and renal function, especially in the elderly, and there has been a controversial relationship between TG levels and renal function. Previous studies have proved that TG levels were significantly associated with renal function^28^. Triglyceride-rich apolipoprotein B-containing lipoproteins may promote the progression of RFD^30^. However, Helsinki Heart Study has provided an evidence that there is no significant association between TG levels and renal function^31^. This study confirmed that TG levels were significantly associated with renal function in Chinese centenarians, and lowering TG therapy may play a role in preserving renal function in addition to preventing cardiovascular disorders.

Smoking is a significant public health problem all over the world, and has been considered to be harmful to renal function in previous studies^32^. Meanwhile, there has been a concern for many countries about the relationship between DM and renal function, and previous large-scale survey in the US general population has concluded that DM was not associated with renal function^33^. Consistent with previous studies, this study demonstrated that with a prevalence of 3.3%, smoking exerted harmful effects on renal function. And with a prevalence of 10.5%, DM had no significant association with renal function in Chinese centenarians.

The current study had one limitation. Smoking was assessed by asking each centenarian whether he or she was a current smoker. Although ex-smoker does not seem to be counted as smoker, it should be considered to be one limitation.

## Conclusion

As the first time all over the world, this study reported low prevalence of MetS and its risk factors, and demonstrated that age, smoking, abdominal obesity (or WC), hypertension (or SBP and DBP) and TG levels were independently associated with renal function in Chinese centenarians. Low prevalence of MetS and its risk factors was a possible reason of longevity, and the interrelationship of age, Mets and its risk factors with renal function was a possible mechanism of longevity in Chinese centenarians. Based on CHCCS, this study not only provided reliable data about Chinese centenarians and analyzed significant relationships between Mets risk factors and renal function, but also explained possible reason and mechanism of longevity.

## Methods

### Study population

CHCCS was performed in population-based individuals aged 100 or above from July 2014 to December 2016 in 18 cities and counties of Hainan Province, China. Its cohort profile has been described previously^34^. Based on National Civil Registry, a total of 1,002 centenarians were identified by Hainan Civil Affairs Bureau and enrolled in this study. Age was ascertained from national identification cards. The following inclusion criteria were used to recruit study participants: (1) was 100 years or older; (2) volunteered to participate in the study and provided written informed consent; and (3) was conscious and could cooperate to complete the home interview, physical examination and blood analysis. The following were participant exclusion criteria: (1) personal identity information was not complete or identification cards showed an age of less than 100 years; (2) refused to comply with the requirements of the study, including the collection of physical or blood samples. There were 874 centenarians included in the final analysis. This study followed the approval from Ethics Committee of Hainan branch of Chinese People’s Liberation Army General Hospital (Sanya, Hainan; Number: 301hn11201601). Written informed consent was obtained from all centenarians in this study. All methods were performed in accordance with the relevant guidelines and regulations.

### Standard procedures

Home interview, physical examination and blood analysis were performed following standard procedures^35^. The research team included internists, geriatricians, cardiologists, endocrinologists, nephrologists and nurses. Smoking was assessed by asking each centenarian whether he or she was a current smoker^36^. Waist circumstance (WC) was measured with a soft tape midway between the lowest rib and the iliac crest. Consistent with current recommendations, systolic and diastolic blood pressures (SBP and DBP) were measured with the right arm of centenarians two times consecutively, with at least 1 minute between measurements, and the reported blood pressures were the average of these two measurements. Samples of venous blood were obtained from the centenarians and transported in chilled bio-transport container (4 °C) to our Central Laboratory within 4 hours. Serum concentrations of fasting blood glucose (FBG), total cholesterol (TC), triglyceride (TG), low-density lipoprotein cholesterol (LDL-C), high-density lipoproteincholesterol (HDL-C) and creatinine were measured using the enzymatic assays (Roche Products Ltd, Basel, Switzerland) on a fully automatic biochemical autoanalyzer (Cobas c702; Roche Products Ltd, Basel, Switzerland). All assays were performed by qualified technicians without knowledge of clinical data.

### Variable definitions

Based on the worldwide consensus on the definition of MetS recommended by International Diabetes Federation, MetS was defined as abdominal obesity plus any two of four additional factors: SBP ≥130 mmHg or DBP ≥85 mmHg (or previously diagnosed hypertension); FBG ≥5.6 mmol/L (or previously diagnosed DM); and TG ≥1.7 mmol/L, HDL-C <1.0 mmol/L in males and <1.3 mmol/L in females (or previously diagnosed dyslipidemia)^37^. Based on Chinese guidelines on prevention and control of obesity, abdominal obesity was defined as WC ≥85 cm for men and ≥80 cm for women^38^. Hypertension was defined as SBP ≥140 mmHg, DBP ≥90 mmHg or taking anti-hypertensive drugs^39^. DM was defined as FBG ≥7.0 mmol/L or taking hypoglycemic drugs/insulin^40^. Dyslipidemia was defined as TG ≥1.7 mmol/L, LDL-C ≥3.37 mmol/L, HDL-C ≥1.04 mmol/L or taking lipid-regulating drugs^41^. Estimated glomerular filtration rate (GFR) was calculated using a modified version of Modification of Diet in Renal Disease (MDRD) equation based on the data from Chinese patients as follows: 175 × serum creatinine (mg/dL)^−1.234^ × age (year)^−0.179^ × 0.79 (if female)^42^.

### Statistical analyses

Continuous variables were described as the mean and standard deviation for variables with normal distribution and the median and interquartile range for variables with skewed distribution. Categorical variables were described as the number and percentage. Continuous variables were compared with Student’s t-test (normal distribution) and Mann–Whitney U test (skewed distribution). Categorical variables were compared with Chi-square test. Pearson’s (continuous variables with normal distribution) and Spearman’s (continuous variables with skewed distribution and categorical variables) correlations were used to assess the simple relationships between MetS risk factors and renal function. In order to assess the independent relationships between MetS risk factors and renal function, multivariate linear regression analyses were adjusted by age, sex, smoking, WC, SBP, DBP, TC, TG, HDL-C, LDL-C and FBG, and multivariate logistic regression analyses were adjusted by age, sex, smoking, abdominal obesity, hypertension, dyslipidemia and DM. Statistical significance was accepted at the two-sided 0.05 level, and confidence interval (CI) was computed at the 95% level. Statistical analyses were performed with Statistic Package for Social Science (SPSS) version 17 (SPSS Inc., Chicago, IL, U.S.).

### Availability of data and materials

In attempt to preserve privacy of patients, clinical data of patients will not be shared; data can be available from authors upon request.
